# Community-acquired *Acinetobacter baumannii* pneumonia: a rare case in Brazil

**DOI:** 10.1590/0037-8682-0301-2022

**Published:** 2022-11-21

**Authors:** Izadora Raduan Brigo, Leandro de Resende Yamamoto, Rodrigo Juliano Molina

**Affiliations:** 1 Universidade Federal do Triângulo Mineiro, Departamento de Clínica Médica, Uberaba, MG, Brasil.

**Keywords:** Acinetobacter baumannii, Community-acquired pneumonia, Alcohol

## Abstract

*Acinetobacter baumannii*, a common pathogen in nosocomial infections, is a rare cause of community-acquired pneumonia. This report highlights the difficulties in its early diagnosis and effective treatment, as it is a multidrug-resistant microorganism with rapid, unfavorable progression. To better understand its clinical outcome, we searched the literature for similar cases but found no community-acquired cases in Brazil.

## INTRODUCTION

*Acinetobacter baumannii* (AB) usually causes nosocomial infections and is a rare cause of community-acquired pneumonia (CAP). It is an aerobic, gram-negative bacillus found in freshwater and soil and may cause a severe and even fatal course if diagnosed and treated late. Symptoms include fever, productive cough, dyspnea, and pleuritic chest pain, which may rapidly progress to acute respiratory failure (ARF)^1^.

Diagnosis is challenging because the gold standard is to isolate the pathogen, which often requires a long time. Therefore, it is associated with a high mortality rate. The most commonly used technique in practice is gram staining to identify gram-negative coccobacilli^2^.

Due to the fulminant clinical course of the infection and its resistance to several classes of antibiotics, the earlier the treatment is started, the better the patient's prognosis. Carbapenems are the main therapeutic option in severe cases, along with aminoglycosides, beta-lactamase inhibitors, and polymyxins^3^. We present the case of a patient diagnosed with CAP that rapidly progressed and died before a late positive culture for AB was reported.

To our knowledge, this is the first report of CAP caused by *AB* in Brazil.

The Research Ethics Committee of the Federal University of Triângulo Mineiro, Minas Gerais, Brazil, approved this case report (Certificado de Apresentação para Apreciação Ética - CAAE: 58470422.4.0000.8667).

## CASE REPORT

**FIGURE 1:**
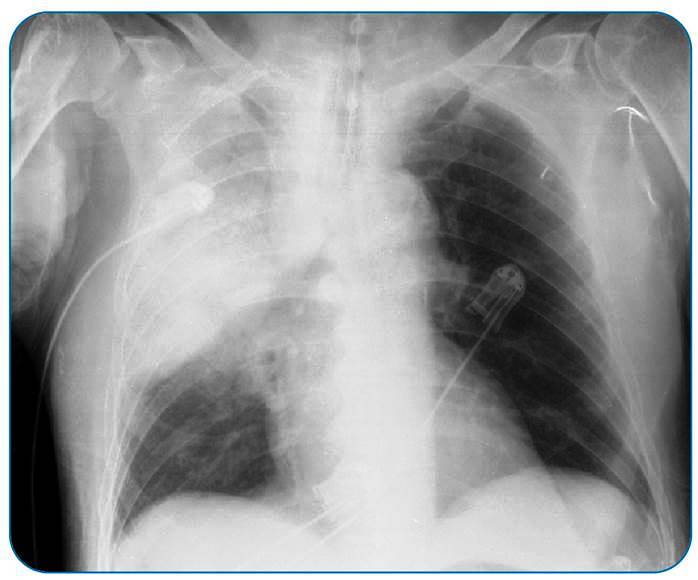
Extensive consolidation in the right upper lobe with air bronchogram and signs of bulging interlobar fissure.

An 82-year-old male with a history of smoking (54 pack-years), alcohol intake, hypertension, prediabetes, and hypothyroidism was admitted with diffuse myalgia, fever, and moderate dyspnea, which progressively worsened. Physical examination revealed peripheral cyanosis, tachycardia, snoring, and diffuse fine rales with desaturation in the ambient air. Within a few hours, the patient developed hemodynamic instability and ARF and required orotracheal intubation. Family members reported staying at a ranch in the previous four days and denied that the patient had been hospitalized or had recently used antibiotics. On admission, the patient presented with acute kidney injury, elevated C-reactive protein (13.4 mg/dL; reference: < 0.1 mg/dL), leukocytosis with left shift, and lactic acidosis. Chest radiography revealed right upper and middle lobar consolidation (Figure 1). Ceftriaxone was initiated; however, the patient maintained severe metabolic acidosis, hemodynamic instability, and difficult ventilation. In less than 24 h, he died, and necropsy was requested to clarify the case. Blood culture (released a few days after death) showed AB growth, and necropsy revealed acute lobar pneumonia as the underlying cause of death.

## DISCUSSION

AB is a gram-negative, multidrug-resistant, aerobic bacillus responsible for several hospital infections, such as sepsis, urinary tract infection, meningitis, and ventilator-associated nosocomial pneumonia. It has a higher incidence in immunosuppressed patients. The prevalence of AB in environmental sources (freshwater, soil, skin, gastrointestinal, and lung secretions in community patients) is quite low^4^.

Its colonization rate is much higher than the infection rate and is considered an uncommon cause of CAP. Cases in subtropical countries, such as Australia and Taiwan (incidence < 10% among all pneumonia of bacterial origin) and in regions with temperate climates are extremely rare^5^.

In recent years, cases of AB pneumonia have been described outside Brazil in patients without recent hospitalization or use of antibiotics, as in the case reported^6^. As it is considered an opportunistic pathogen that affects immunocompromised patients, it is rarely found in community infections. In this case, an association between smoking, alcoholism, diabetes mellitus, and chronic obstructive pulmonary disease was recorded^4^. Therefore, CAP by AB should be suspected in admitted patients who are critically ill because it can lead to sepsis, acute respiratory distress syndrome, and disseminated intravascular coagulation in a higher proportion than that resulting from nosocomial pneumonia caused by the same pathogen².

Cases of CAP caused by AB are mainly reported in tropical and subtropical countries during the summer months when temperatures and humidity are higher. The symptoms appear acutely and become fulminant within approximately 48-72 h in the presence of productive cough, with or without hemoptysis, dyspnea, ventilator-dependent chest pain, and fever. On chest radiography, the right lung was predominantly involved, although the infiltrate was bilateral, with the appearance of acute respiratory distress syndrome. Its rapid development into septic shock with multiple organ failure associated with diagnostic difficulty contributes to its high mortality rate (40-64%)^1^ ^,^ ^6^.

Studies have already shown that alcoholism and cancer may lead to fulminant CAP, which presents with normal leukocytes or leukopenia, similar to pneumonia caused by other pathogens such as *Klebsiella pneumoniae* and *Streptococcus pneumoniae* ^5^. In respiratory tract infections, innate host defense mechanisms are characterized by the activation of phagocytic cells, such as macrophages, which are responsible for detecting and eliminating aggressive microorganisms when acquired immunity is activated. During infection by AB, alveolar macrophages are stimulated to produce proinflammatory cytokines that promote the recruitment of neutrophils, in addition to the production of high levels of nitric oxide to fight the pathogen. However, alcohol consumption compromises the immune response against AB, as it leads to dysfunction of alveolar macrophages^7^.

The diagnosis of AB-related CAP is challenging because of its rarity in Brazil and worldwide, raising an alert for it to be considered a diagnostic hypothesis in cases of severe CAP, along with other more prevalent pathogens. The gold standard is the isolation of the pathogen, and culture is the most common method; however, it takes weeks for the result, which is unfavorable given the fulminant course of the disease^1^. In contrast, sputum gram stain is considered useful, fast, and inexpensive for early diagnosis, in which AB appears as gram-negative coccobacilli, with a sensitivity of 15-100% and a specificity of 11-100%².

Because of its fulminant course, the prognosis of CAP caused by AB depends on the early administration of antibiotic therapy, which can reduce the mortality rate by up to 11% with appropriate treatment^1^. In the mid-1970s, AB was susceptible to several antibiotics; however, its resistance has increased considerably in the last decade^8^.

Mechanisms include the loss of porin channels that assist in transporting antimicrobials within cells, reduced access to bacterial targets, and mutations that alter bacterial function by decreasing their affinity for antibiotics^9^.

Even so, carbapenems remain the treatment of choice, but resistance to this class of antibiotics has been increasing substantially. Ampicillin (beta-lactam) is not active against Acinetobacter spp.; only sulbactam (beta-lactamase inhibitor) is, in combination with ampicillin/sulbactam. Amikacin and tobramycin (aminoglycosides) are rarely used because of their toxicity, and colistin or polymyxin E/B are used to treat multidrug-resistant, gram-negative bacteria^3^.

An article published in 2018 by the Infectious Disease Society of America presented 19 cases of CAP-AB in Southeast Asia, Australia, and North America. Of these, 17 progressed to fulminant disease, similar to our case. To our knowledge, CAP-AB has not been reported in Brazil in databases such as PubMed Central (PMC), PubMed, SciELO, Cochrane Library, and Virtual Health Library. Thus, this case highlights the possibility of unusual bacteria, such as AB, causing CAP in older patients with comorbidities. It is essential to demonstrate its severe and fulminant clinical course to reduce the underreporting of such cases. Additionally, Brazil is a country that favors infection by this pathogen due to its climatic conditions (hot and humid climate)^6^.
